# Anti-Fibrotic Effect of Losartan, an Angiotensin II Receptor Blocker, Is Mediated through Inhibition of ER Stress via Up-Regulation of SIRT1, Followed by Induction of HO-1 and Thioredoxin

**DOI:** 10.3390/ijms18020305

**Published:** 2017-01-31

**Authors:** Hyosang Kim, Chung Hee Baek, Raymond Bok Lee, Jai Won Chang, Won Seok Yang, Sang Koo Lee

**Affiliations:** Division of Nephrology, Department of Internal Medicine, Asan Medical Center, Asan Institute for Life Sciences, University of Ulsan, 88, Olympic-ro 43-gil, Songpa-gu, Seoul 138-736, Korea; mateus@amc.seoul.kr (H.K.); bch393@naver.com (C.H.B.); leerb@umich.edu (R.B.L.); jwchang@amc.seoul.kr (J.W.C.); wsyang@amc.seoul.kr (W.S.Y.)

**Keywords:** ER stress, HO-1, losartan, SIRT1, thioredoxin

## Abstract

Endoplasmic reticulum (ER) stress is increasingly identified as modulator of fibrosis. Losartan, an angiotensin II receptor blocker, has been widely used as the first choice of treatment in chronic renal diseases. We postulated that anti-fibrotic effect of losartan is mediated through inhibition of ER stress via SIRT1 (silent mating type information regulation 2 homolog 1) hemeoxygenase-1 (HO-1)/thioredoxin pathway. Renal tubular cells, tunicamycin (TM)-induced ER stress, and unilateral ureteral obstruction (UUO) mouse model were used. Expression of ER stress was assessed by Western blot analysis and immunohistochemical stain. ER stress was induced by chemical ER stress inducer, tunicamycin, and non-chemical inducers such as TGF-β, angiotensin II, high glucose, and albumin. Losartan suppressed the TM-induced ER stress, as shown by inhibition of TM-induced expression of GRP78 (glucose related protein 78) and p-eIF2α (phosphospecific-eukaryotic translation initiation factor-2α), through up-regulation of SIRT1 via HO-1 and thioredoxin. Losartan also suppressed the ER stress by non-chemical inducers. In both animal models, losartan reduced the tubular expression of GRP78, which were abolished by pretreatment with sirtinol (SIRT1 inhibitor). Sirtinol also blocked the inhibitory effect of losartan on the UUO-induced renal fibrosis. These findings provide new insights into renoprotective effects of losartan and suggest that SIRT1, HO-1, and thioredoxin may be potential pharmacological targets in kidney diseases under excessive ER stress condition.

## 1. Introduction

Renal fibrosis, particularly tubulointerstitial fibrosis, is considered to be a central event in the progression of chronic kidney diseases, regardless of the original cause of the kidney disease. Therefore, renal fibrosis is a reliable predictor and a major determinant of renal insufficiency. However, the underlying mechanism has not been completely understood. The endoplasmic reticulum (ER) stress refers to physiological or pathological states that result in accumulation of misfolded proteins in the ER, which leads to cell stress conditions (ER stress). To mitigate protein misfolding stress, cells activate a homeostatic intracellular signaling network cumulatively called the unfolded protein response (UPR). UPR reestablishes the homeostasis of ER through the activation of three major sensors known as PERK (PKR-like ER kinase), IRE-1 (inositol requiring enzyme-1), and ATF-6 (activating transcription factor 6). The ER chaperone GRP78 (glucose related protein 78) is bound to these three transmembrane sensors. Under ER stress conditions, GRP78 dissociates from the ER stress sensors and binds to misfolded proteins, which leads to activation of these sensors and initiation of the UPR. Therefore, GRP78 has been referred to as a central regulator of ER homeostasis. Although ER stress serves a protective role that allows cells to survive from the noxious stimuli, excessive ER stress triggers apoptosis through CHOP (C/EBP homologous protein) induced by PERK and ATF6 pathways [1,2]. Furthermore, ER stress and UPR are known to contribute to other ER stress-independent cellular responses [3].Therefore, sustained ER stress has been implicated in the development and progression of many chronic diseases [4].

Recently, ER stress has been identified as an important factor in the development of organ fibrosis through activation of pro-apoptotic pathways, induction of epithelial-mesenchymal transition, and promotion of inflammatory responses [5,6]. Thus, inhibition of ER stress may serve as one of the possible therapeutic approaches to organ fibrosis and inflammation. Angiotensin converting enzyme (ACE) inhibitors and angiotensin II receptor blockers (ARBs) are widely used drugs to reduce proteinuria and to attenuate the renal tubulointerstitial fibrosis through inhibition of angiotensin II. In addition to its hemodynamic effects, angiotensin II is also known to be involved in various pro-inflammatory actions including induction of TGF-β, adhesion molecules, chemokines, cytokines, and reactive oxygen species (ROS) generation [7,8]. Therefore, ACE inhibitors and ARBs have been recommended as the first choice of treatment in chronic renal diseases. However, it remains unclear whether ACE inhibitors or ARBs can suppress the ER stress in renal tubular epithelial cells.

Sirtuins are a family of NAD^+^ (nicotinamide adenine dinucleotide)-dependent protein deacetylases that exert multiple cellular functions by interacting with a wide range of signaling molecules, transcription factors, histones, and enzymes [9]. Mammals have seven different sirtuins assigned as SIRT1-7. SIRT1 (silent mating type information regulation 2 homolog 1) is known to exert a renoprotective effect by inhibiting interstitial fibrosis, inflammation, and apoptosis through deacetylation or interaction with several target proteins [10]. Recently, it has been reported that up-regulation of SIRT1 is able to inhibit the ER stress in renal tubular epithelial cells [11].

Heme oxygenase-1 (HO-1) is a highly inducible isoform enzyme that degrades heme into biliverdin, carbon monoxide (CO), and iron, which have anti-oxidant, anti-apoptotic, and anti-inflammatory properties [12]. Thioredoxin is a 12-kD oxidoreductase enzyme that acts as an antioxidant by facilitating the reduction of other proteins by cysteine thiol-disulfide exchange [13]. It has been shown that two antioxidant systems can protect cells from reactive oxygen species (ROS)-induced oxidative damage. In addition, HO-1 and thioredoxin have been implicated as modulators in inhibition of ER stress [14,15].

However, the potential effects of ACE inhibitors or ARBs on the expression of SIRT1, HO-1, and thioredoxin have not been well studied. Since ACE inhibitors and ARBs are drugs commonly prescribed for the prevention of progression of kidney injury, it would be interesting to identify the effects of ACE inhibitors or ARBs on ER stress, SIRT1, HO-1, and thioredoxin.

We postulated that the anti-fibrotic effect of ARBs might be mediated through the inhibition of ER stress via up-regulation of SIRT1, followed by induction of HO-1 and thioredoxin in renal tubular cells. We examined the effects of losartan, a classic drug of ARBs, on the ER stress induced by both chemical inducers, such as tunicamycin, and non-chemical inducers, such as tumor growth factor-β (TGF-β), angiotensin II, high glucose, and albumin in tubular HK-2 cells. We also examined whether the inhibitory effect of losartan on ER stress was mediated through up-regulation of SIRT1 via induction of HO-1 and thioredoxin. We further investigated the in vivo effects of losartan with or without sirtinol (an inhibitor of SIRT1) on ER stress and renal fibrosis using a tunicamycin-induced ER stress mouse model and a unilateral ureteral obstruction mouse model.

## 2. Results

### 2.1. Losartan Suppressed the ER Stress Induced by Tunicamycinin in Tubular Epithelial Cells

**Figure 1:**
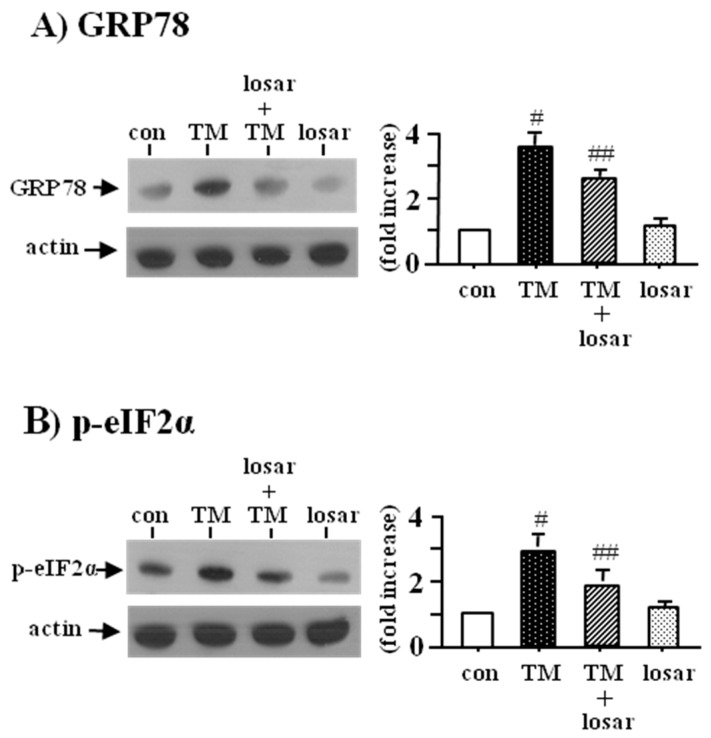
Inhibition of tunicamycin-induced endoplasmic reticulum (ER) stress by losartan. Proximal tubular cells (HK-2 cells) were incubated with tunicamycin (TM, 0.2 µM) with or without losartan (losar, 10 µM) for 24 h. Expression of GRP78 (**A**) and p-eIF2α (**B**) was examined by Western blot analysis. The relative densities of the bands for GRP78 (glucose related protein 78) and p-eIF2α (phosphospecific-eukaryotic translation initiation factor-2α) were normalized to those for actin for standardization. Representative blots and quantitative analysis from three independent experiments were shown. Results were expressed as *n*-fold increase over control as mean ± S.E. #: *p* < 0.05 vs. con (control), ##: *p* < 0.05 vs. TM.

To determine whether losartan suppressed the tunicamycin-induced ER stress in tubular HK-2 cells, we examined the change of two ER stress biomarkers, the up-regulation of GRP78 and p-eIF2α. Losartan (10 µM) suppressed the tunicamycin-induced ER stress, as shown by inhibition of tunicamycin-induced up-regulation of GRP78 and p-eIF2α (*p* < 0.05). Losartan itself had no effects on the expression of GRP78 and p-eIF2α (Figure 1).

### 2.2. Inhibitory Effect of Losartan on the Tunicamycin-Induced ER Stress Was Mediated through Up-Regulation of SIRT1

**Figure 2:**
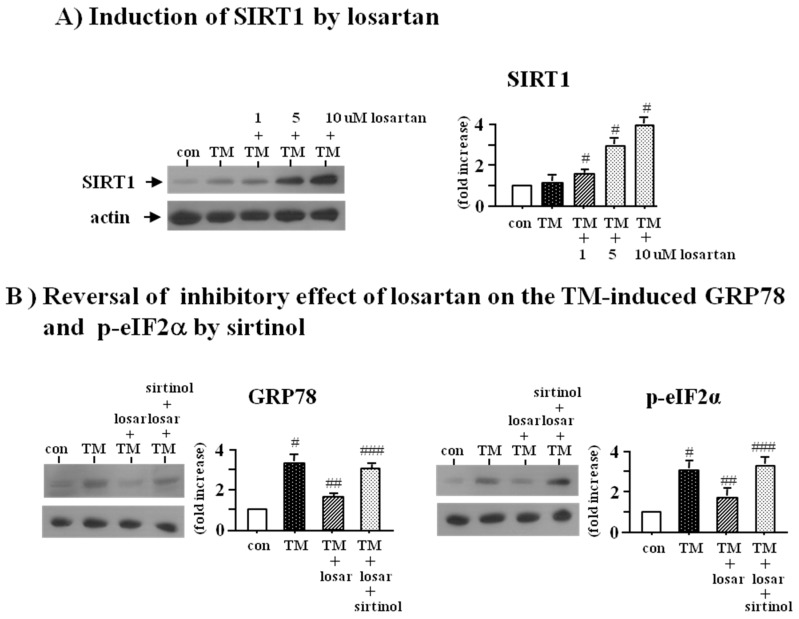
Induction of SIRT1 (silent mating type information regulation 2 homolog 1) by losartan (**A**) and reversal of inhibitory effect of losartan on the tunicamycin-induced ER stress by sirtinol (**B**). Proximal tubular cells were incubated with tunicamycin (TM, 0.2 µM) with or without losartan (losar, 1–10 µM) for 24 h. Expression of SIRT1 was examined by Western blot analysis (**A**); Proximal tubular cells were incubated with tunicamycin (TM, 0.2 µM) with or without losartan (losar, 10 µM) and sirtinol (SIRT1 inhibitor, 10 µM) for 24 h. Expression of GRP78 and p-eIF2α was examined by Western blot analysis (**B**). The relative densities of the bands for SIRT1, GRP78, and p-eIF2α were normalized to those for actin for standardization. Representative blots and quantitative analysis from three independent experiments were shown. Results were expressed as *n*-fold increase over control as mean ± S.E. #: *p* < 0.05 vs. con (control); ##: *p* < 0.05 vs. TM; ###: *p* < 0.05 vs. TM + losar.

Losartan induced the expression of SIRT1 in a dose dependent manner (1–10 µM) (Figure 2A). To determine whether up-regulation of SIRT1 by losartan was directly involved in losartan’s inhibitory effect on the tunicamycin-induced ER stress, we examined the effect of SIRT1 inhibitor (sirtinol, 10 µM). Western blot analysis revealed that sirtinol blocked the inhibitory effect of losartan on the tunicamycin-induced ER stress (*p* < 0.05), suggesting that the inhibitory effect of losartan on the ER stress was mediated through up-regulation of SIRT1 (Figure 2B).

### 2.3. Inhibitory Effect of Losartan on the Tunicamycin-Induced ER Stress Was Mediated through Induction of Heme Oxygenase-1 and Thioredoxin

**Figure 3:**
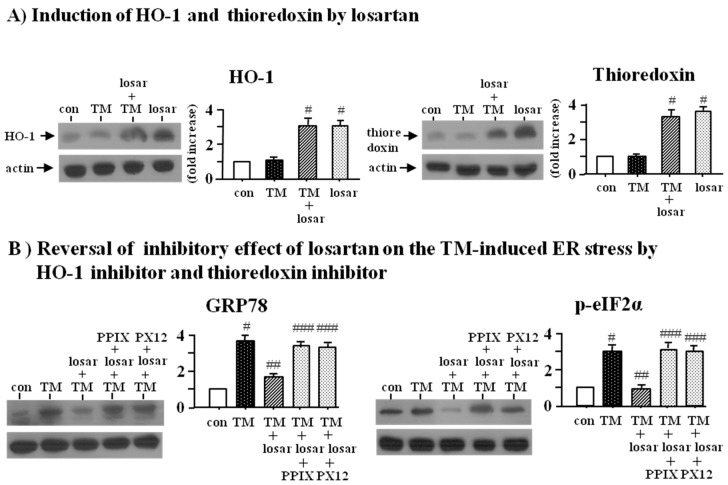
Induction of HO-1 (hemeoxygenase-1) and thioredoxin by losartan (**A**) and reversal of inhibitory effect of losartan on the tunicamycin-induced ER stress by HO-1 inhibitor (PPIX) and thioredoxin inhibitor (PX12) (**B**). Proximal tubular cells were incubated with tunicamycin (TM, 0.2 µM) with or without losartan (losar, 10 µM) for 24 h. Expression of heme oxygenase-1 (HO-1) and thioredoxin was examined by Western blot analysis (**A**). Proximal tubular cells were incubated with tunicamycin (TM, 0.2 µM) with or without losartan (losar, 10 µM), heme oxygenase-1 inhibitor (PPIX, 20 µM), and thioredoxin inhibitor (PX12, 25 µM) for 24 h. Expression of GRP78 and p-eIF2α was examined by Western blot analysis (**B**). The relative densities of the bands for HO-1, thioredoxin, GRP78, and p-eIF2α were normalized to those for actin for standardization. Representative blots and quantitative analysis from three independent experiments were shown. Results were expressed as *n*-fold increase over control as mean ± S.E. #: *p* < 0.05 vs. con (control); ##: *p* < 0.05 vs. TM; ###: *p* < 0.05 vs. TM + losar.

Losartan (10 µM) increased the expression of heme oxygenase (HO-1) and thioredoxin (Figure 3A). To determine whether up-regulation of HO-1 and thioredoxin by losartan was directly involved in losartan’s inhibitory effect on the tunicamycin-induced ER stress, we examined the effect of HO-1 inhibitor (Zinc protoporphyrin IX, Zn(II)PPIX, 10 µM) and thioredoxin inhibitor (PX12, 20 µM). Western blot analysis revealed that both Zn(II)PPIX and PX12 reversed losartan’s inhibitory effect on the tunicamycin-induced ER stress (*p* < 0.05), suggesting that the inhibitory effect of losartan on the ER stress was mediated through induction of HO-1 and thioredoxin (Figure 3B).

### 2.4. Inhibitory Effect of Losartan on the Tunicamycin-Induced ER Stress Was Mediated through Up-Regulation of SIRT1, Followed by Induction of HO-1 and Thioredoxin

**Figure 4:**
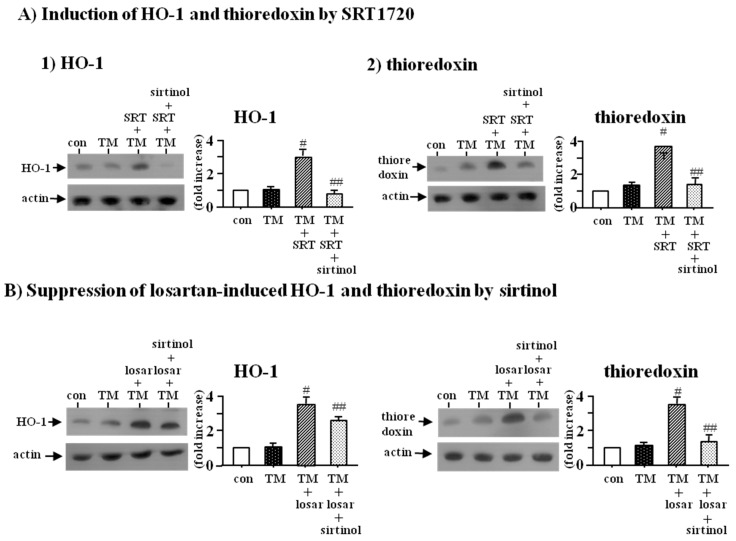
Induction of HO-1 and thioredoxin by SRT1720 (**A**) and suppression of losartan-induced HO-1 and thioredoxin by sirtinol (**B**). Proximal tubular cells were incubated with tunicamycin (TM, 0.2 µM) with or without SRT1720 (SRT, SIRT1 inducer, 2.5 µM) and sirtinol (SIRT1 inhibitor, 10 µM) for 24 h (**A**); Proximal tubular cells were incubated with tunicamycin (TM, 0.2 µM) with or without losartan (losar, 10 µM) and sirtinol (SIRT1 inhibitor, 10 µM) for 24 h (**B**). Expression of heme oxygenase-1 (HO-1) and thioredoxin was examined by Western blot analysis. The relative densities of the bands for HO-1 and thioredoxin were normalized to those for actin for standardization. Representative blots and quantitative analysis from three independent experiments were shown. Results were expressed as *n*-fold increase over control as mean ± S.E. #: *p* < 0.05 vs. con (control) or TM; ##: *p* < 0.05 vs. TM + SRT or TM + losar.

To determine whether SIRT1 could induce the expression of HO-1 and thioredoxin, we examined the effect of SRT1720 (SIRT1 inducer, 2.5 µM) with or without sirtinol (SIRT1inhibitor, 10 µM). Western blot analysis showed that SRT1720 increased the expression of HO-1 and thioredoxin, which were prevented by sirtinol (*p* < 0.05) (Figure 4A).

To confirm whether induction of HO-1 and thioredoxin by losartan was mediated through up-regulation of SIRT1, we examined the effect of sirtinol (SIRT1 inhibitor, 10 µM). Western blot analysis revealed that sirtinol blocked the losartan-induced expression of HO-1 and thioredoxin (*p* < 0.05) (Figure 4B). These data indicated that the inhibitory effect of losartan on the tunicamycin-induced ER stress was mediated through up-regulation of SIRT1, followed by induction of HO-1 and thioredoxin.

### 2.5. Losartan also Inhibited the Tunicamycin-Induced Epithelial-Mesenchymal Transition (EMT)

**Figure 5:**
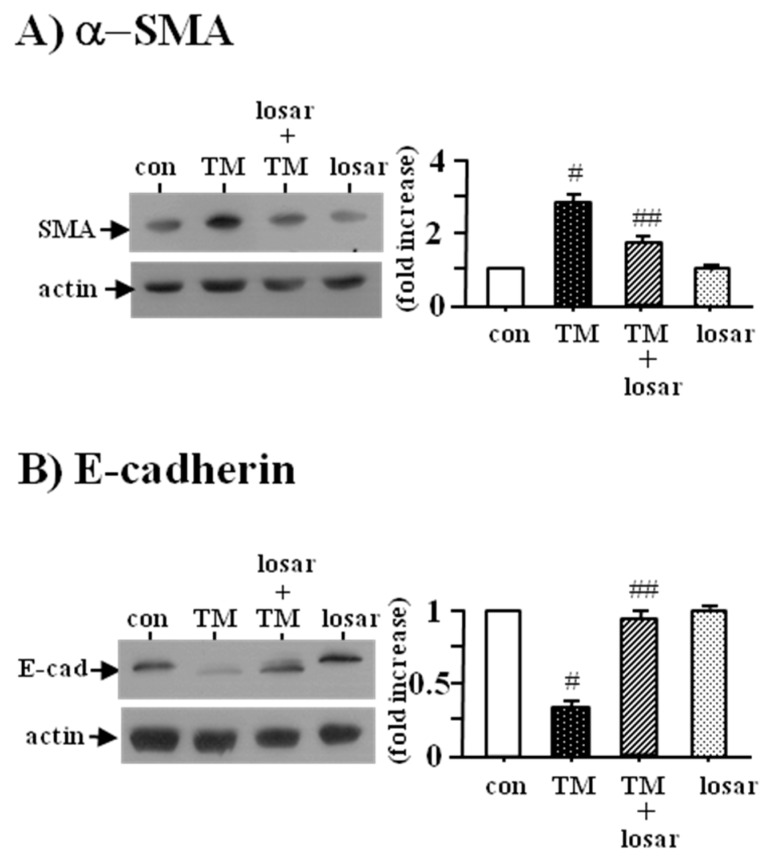
Inhibition of tunicamycin-induced epithelial mesenchymal transition by losartan. Proximal tubular cells were incubated with tunicamycin (TM, 0.2 µM) with or without losartan (losar, 10 µM) for 24 h. Expression of α-SMA (α-smooth muscle actin) (**A**) and E-cadherin (**B**) was examined by Western blot analysis. The relative densities of the bands for α-SMA and E-cadherin were normalized to those for actin for standardization. Representative blots and quantitative analysis from three independent experiments were shown. Results were expressed as *n*-fold increase over control as mean ± S.E. #: *p* < 0.05 vs. con (control); ##: *p* < 0.05 vs. TM.

To determine whether losartan inhibited the tunicamycin-induced EMT, we examined the change of two EMT biomarkers, the up-regulation of α-smooth muscle actin and the down-regulation of E-cadherin. Losartan (10 µM) suppressed the tunicamycin-induced EMT, as evidenced by the inhibition of tunicamycin-induced up-regulation of α-smooth muscle actin and the down-regulation of E-cadherin (*p* < 0.05). Losartan itself had no effects on the expression of α-smooth muscle actin and E-cadherin (Figure 5).

### 2.6. Treatment with Losartan Reduced the Renal Tubular Expression of GRP78 and Increased the Expression of HO-1 and Thioredoxin through the Up-Regulation of SIRT1 in a Mouse Model of Tunicamycin-Induced ER Stress

**Figure 6:**
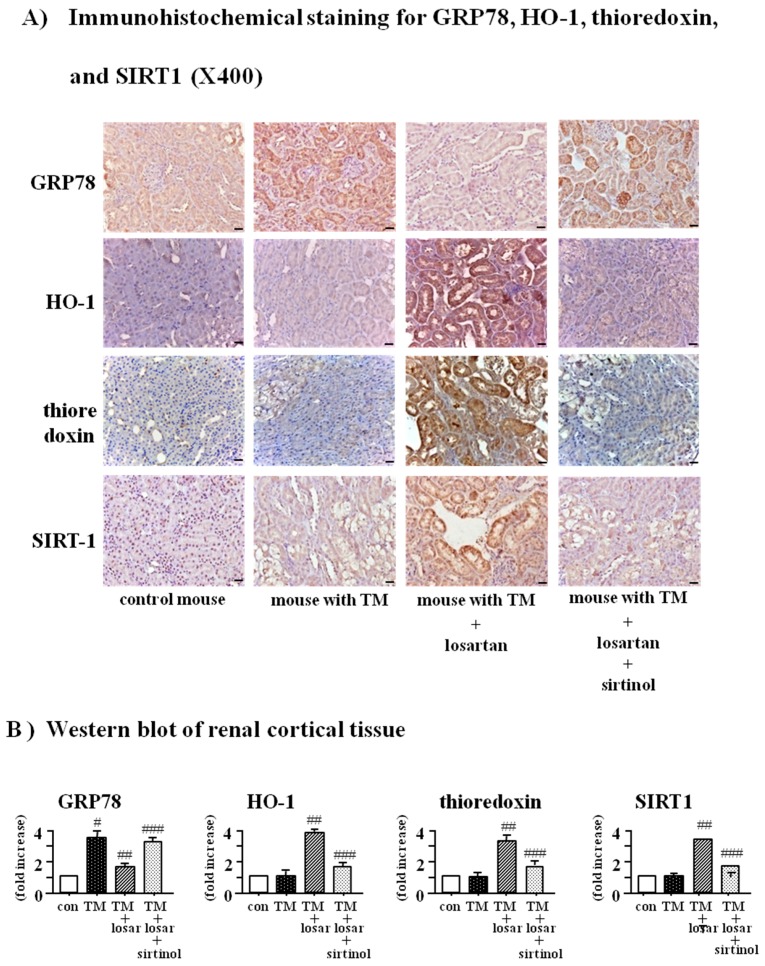
Reduction of renal tubular GRP78 expression and induction of HO-1 and thioredoxin by losartan through up-regulation of SIRT1 in a mouse model of tunicamycin-induced ER stress. Tunicamycin-induced ER stress mouse model was induced by a single intraperitoneal injection of tunicamycin (TM, 2 mg/kg). Mice were randomly divided into four groups: control mice (*n* = 4), mice with TM injection (TM mouse, *n* = 4), TM mice with losartan (*n* = 4), and TM mice with losartan plus sirtinol (SIRT1 inhibitor, 5 mg/kg intraperitoneally). (*n* = 4). Losartan was administered by gavage at 10 mg/kg/day. Immunohistochemical staining (**A**) and Western blot of renal cortical tissue (**B**) for GRP78, heme oxygenase-1 (HO-1), thioredoxin, and SIRT1 were performed. Representative microscopic scans and quantitative analysis were shown. #: *p* < 0.05 vs. con (control); ##: *p* < 0.05 vs. TM; ###: *p* < 0.05 vs. TM + losartan (losar); scale bars = 20 µm.

To demonstrate in vivo inhibitory the effect of losartan on the tunicamycin-induced ER stress, we performed an animal study using a mouse model of tunicamycin-induced ER stress. Consistent with the results of in vitro culture study, treatment with losartan reduced the renal tubular expression of GRP78 and increased HO-1, thioredoxin, and SIRT1, which were abolished by pretreatment with sirtinol (SIRT1 inhibitor, 10 µM) in a mouse model of tunicamycin-induced ER stress (*p* < 0.05) (Figure 6A). Western blot of renal cortical tissue showed the same results (Figure 6B).

### 2.7. Losartan also Suppressed the ER Stress Induced by Non-Chemical ER Stress Inducers Such as Tumor Growth Factor-β (TGF-β), Angiotensin II, High Glucose, and Albumin through Induction of SIRT1

**Figure 7:**
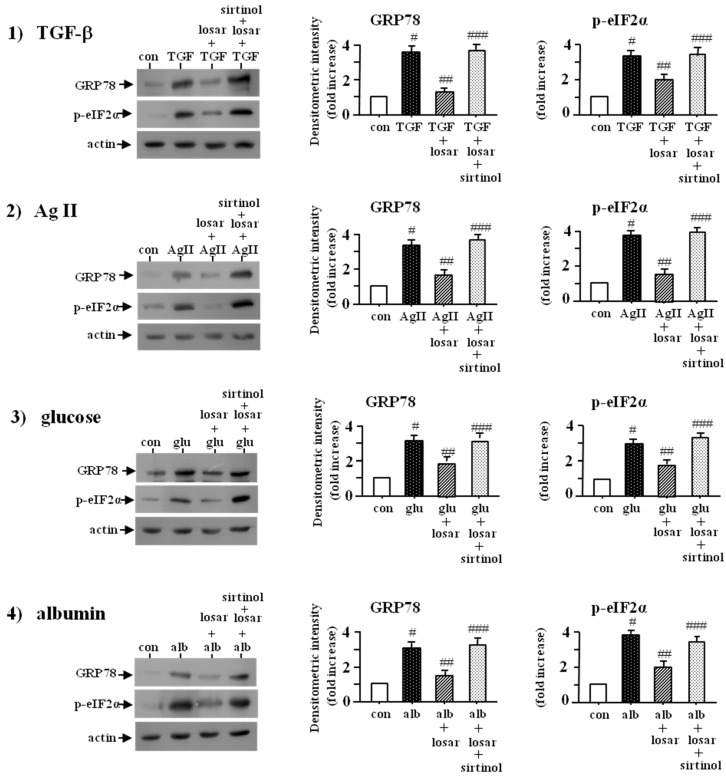
Inhibition of TGF-β-, angiotensin II-, high glucose-, and albumin-induced ER stress by losartan, which were blocked by sirtinol. Proximal tubular cells were incubated with (tumor growth factor-β) TGF-β (10 ng/mL), angiotensin II (AgII, 1 µM), high glucose (glu, 30 mM), and albumin (alb, 5 mg/mL) for three days and then treated with or without losartan (losar, 10 µM) and sirtinol (SIRT1 inhibitor, 10 µM) for two days. Expression of GRP78 and p-eIF2α was examined by Western blot analysis. The relative densities of the bands for GRP78 and p-eIF2α were normalized to those for actin for standardization. Representative blots and quantitative analysis from three independent experiments were shown. Results were expressed as *n*-fold increase over control as mean ± S.E. #: *p* < 0.05 vs. con (control); ##: *p* < 0.05 vs. TGF-β; AgII, glu or alb; ###: *p* < 0.05 vs. TGF-β + losar, AgII + losar, glu + losar, or alb + losar.

To determine whether losartan suppressed the TGF-β-, angiotensin II-, high glucose-, and albumin-induced ER stress, we examined the change of two ER stress biomarkers, the up-regulation of GRP78 and p-eIF2α. Losartan (10 µM) suppressed the TGF-β-, angiotensin II-, high glucose-, and albumin-induced ER stress, as shown by inhibition of up-regulation of GRP78 and p-eIF2α expression, which were abolished by pretreatment with sirtinol (SIRT1 inhibitor, 10 µM) (*p* < 0.05) (Figure 7).

### 2.8. Treatment with Losartan Reduced the Renal Tubular Expression of GRP78 and Renal Fibrosis through the Up-Regulation of SIRT1 in a Mouse Model of Unilateral Ureteral Obstruction (UUO)

**Figure 8:**
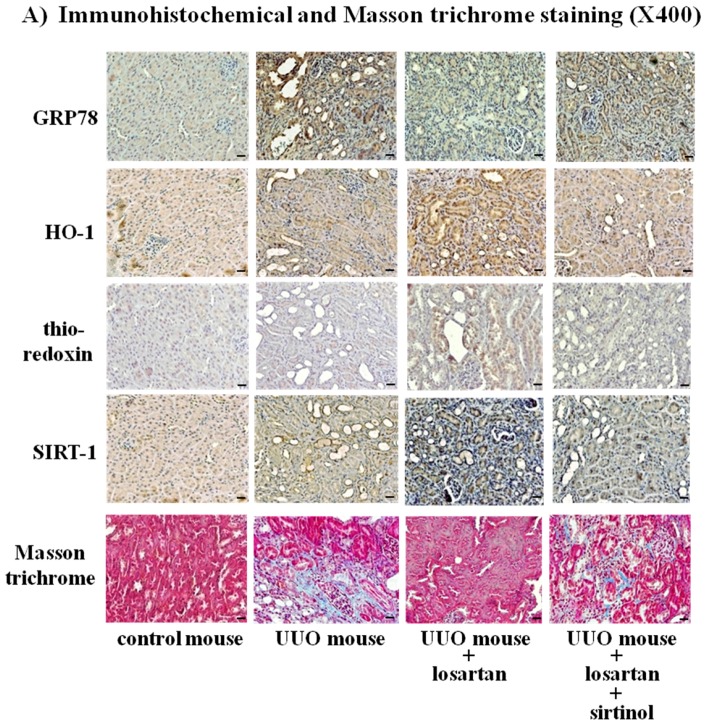

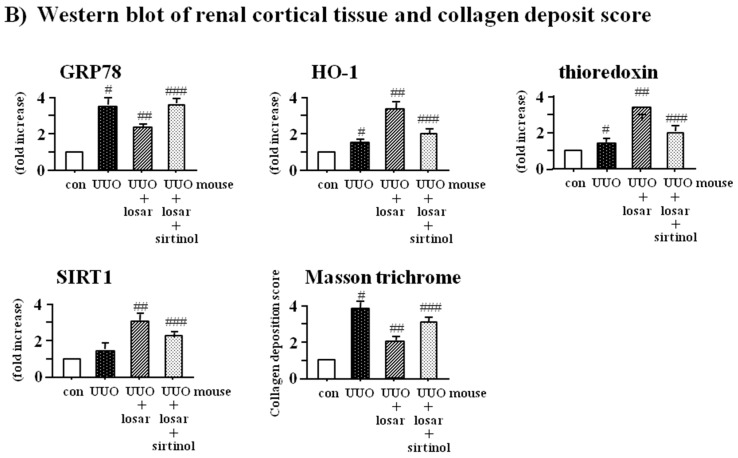
Reduction of renal tubular GRP78 expression and renal fibrosis by losartan through up-regulation of SIRT1 in a mouse model of unilateral ureteral obstruction. Unilateral ureteral obstruction (UUO) mouse model was induced by ligation of left ureter through flank incision. Mice were randomly divided into four groups: control mice (*n* = 4), mice with UUO (UUO mouse) (*n* = 4), UUO mice with losartan (*n* = 4), and UUO mice with losartan plus sirtinol (SIRT1 inhibitor, 5 mg/kg i.p.) (*n* = 4). Losartan (losar) was administered by gavage at 10 mg/kg/day. Immunohistochemical and Masson trichrome staining (**A**) and Western blot of renal cortical tissue and collagen deposit score (**B**) were performed. Representative microscopic scans and quantitative analysis were shown. #: *p* < 0.05 vs. con (control); ##: *p* < 0.05 vs. UUO; ###: *p* < 0.05 vs. UUO + losar; scale bars = 20 µm.

To demonstrate the in vivo inhibitory effect of losartan on the UUO-induced ER stress and renal fibrosis, we performed an animal study using a mouse model of UUO. Treatment with losartan reduced the renal tubular expression of GRP78 and increased the expression of HO-1, thioredoxin, and SIRT1, which were abolished by sirtinol in a mouse model of UUO (*p* < 0.05). Furthermore, sirtinol blocked the inhibitory effect of losartan on the UUO-induced renal fibrosis (*p* < 0.05) (Figure 8A). Western blot of renal cortical tissue and collagen deposit score showed the same results (Figure 8B).

## 3. Discussion

**Figure 9:**
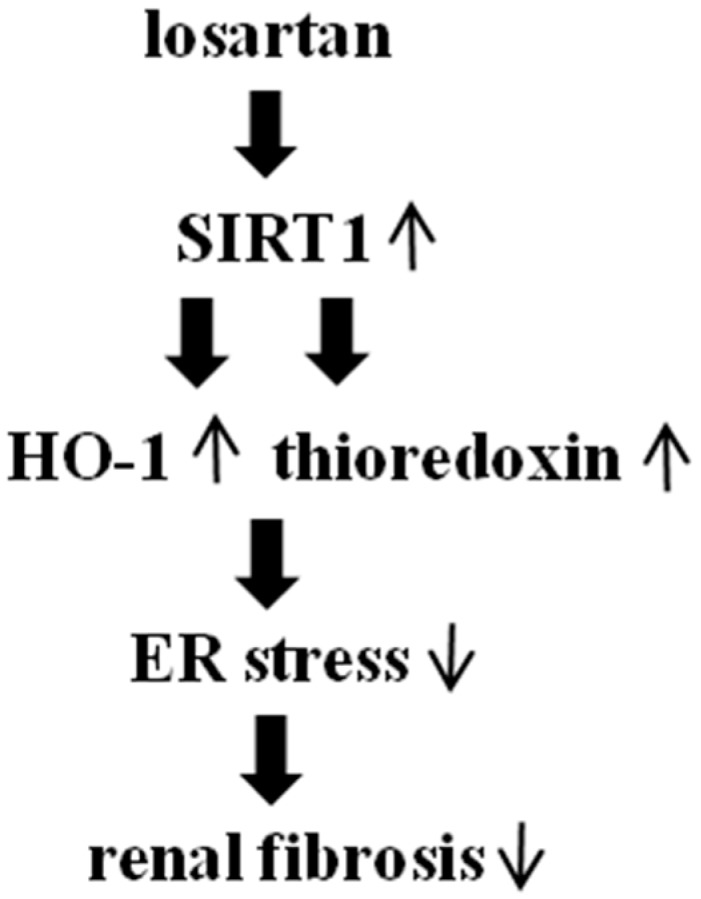
Proposed signaling pathways involved in anti-fibrotic effect of losartan. ↑ represents up-regulation; ↓ represents down-regulation.

The present study demonstrated that losartan suppressed the tunicamycin (TM)-induced ER stress through up-regulation of SIRT1, followed by induction of heme oxygenase-1 and thioredoxin in tubular epithelial cells. Losartan also reduced the ER stress induced by non-chemical agents such as TGF-β, angiotensin II, high glucose, and albumin. In addition, losartan inhibited the TM-induced epithelial mesenchymal transition. Consistent with the results of the cell culture study, losartan reduced the renal tubular expression of GRP78 and increased the expression of SIRT1, HO-1, and thioredoxin in a mouse model of tunicamycin-induced ER stress and unilateral ureteral obstruction (UUO), which were abolished by pretreatment with sirtinol (SIRT1 inhibitor). Sirtinol also blocked the inhibitory effect of losartan on the UUO-induced renal fibrosis, suggesting that the anti-fibrotic effect of losartan might be mediated at least in part through inhibition of ER stress via up-regulation of SIRT1, followed by induction of HO-1 and thioredoxin (Figure 9).

Tubulointerstitial fibrosis plays a most important role in the progression of chronic renal disease to end-stage renal failure. Losartan is an angiotensin II receptor blocker (ARB) that is widely used to delay the progression of chronic renal disease. However, the mechanism underlying the renoprotective effects of losartan has not been revealed completely.

Recently, ER stress and unfolded protein response (UPR) pathways are emerging as important factors in the development of organ fibrosis and inflammation [5,6]. Inhibition of ER stress by 4-phenylbutyric acid, an ER stress inhibitor, was reported to suppress the cardiac and renal fibrosis in animal models [16,17,18]. Furthermore, ER stress is also known to elicit inflammatory responses through activation of NF-κB and NLRP3 inflammasome, induction of cytokines such as IFN-β, IL-23, endothelin-1, and release of TGF-β and collagen I [19,20,21,22,23,24].

Although a role of ER stress and UPR activation in kidney disease is increasingly convincing, the potential effect of losartan on the ER stress in renal tubular epithelial cells has not been analyzed in detail.

In this study, we found that losartan suppressed the ER stress induced by chemical ER stress inducers (tunicamycin) as well as non-chemical inducers such as TGF-β, angiotensin II, high glucose, and albumin in renal tubular epithelial cells. Consistent with the results of the cell culture study, animal studies showed that losartan reduced the tubular expression of ER stress protein (GRP78) in a mouse model of tunicamycin-induced ER stress and unilateral ureteral obstruction (UUO). In agreement with our study, it has been reported that losartan is able to reduce the glucose-induced ER stress in pancreatic β cells [3]. Similarly, animal studies also showed that treatment with other ARBs such as candesartan and olmesartan could prevent the progression of dilated cardiomyopathy in rats through the inhibition of ER stress and reduce the ER stress-induced apoptosis in streptozotocin-induced diabetic animal model, respectively [25,26]. Together with these reports, our data suggested that inhibition of ER stress could be one of the possible mechanisms underlying the renoprotective effect of ARBs. SIRT1, a NAD-dependent protein deacetylase, contributes to regulate intracellular metabolism and attenuate reactive oxidative species (ROS)-induced apoptosis leading to longevity and acute stress resistance [9]. In kidney, SIRT1 is reported to exhibit renoprotection through the reduction of fibrosis, anti-apoptotic effect, anti-inflammatory effect, and induction of autophagy [10]. In addition, SIRT1 is known to suppress the ER stress in renal tubular cells [11]. However, the effect of losartan on the expression of SIRT1 in proximal tubular cells has not been explored so far.

We found that losartan could induce the expression of SIRT1 in a dose dependent manner in renal tubular cells. Animal studies also showed that treatment with losartan induced the tubular expression of SIRT1. In support of our results, it has been reported that treatment with other ARBs, such as telmisartan and valsartan, increased the expression of SIRT1 in skeletal muscle cells of obese db/db mice and in myocardial cells of type-2 diabetes mellitus mice, respectively [27,28]. In addition, we also found that the inhibitory effect of losartan on the ER stress was mediated through the up-regulation of SIRT1. Heme oxygenase-1 (HO-1) is the key rate-limiting enzyme in heme degradation, which produces biliverdin, carbon monoxide (CO), and iron. Clearance of excess free heme by HO-1 is critical in preventing heme-induced production of reactive oxygen species (ROS). Furthermore, CO and biliverdin is known to have anti-oxidant, anti-apoptotic, and anti-inflammatory properties, resulting in cytoprotection. CO also attenuates the ER stress-induced activation of IRE1, ATF6, and CREBH [10].

Thioredoxin is an important endogenous antioxidant and is ubiquitously expressed [13]. Thioredoxin is also known as an endogenous inhibitor of ASK1, which mediates apoptosis on ER stress [29]. Zeng et al. demonstrated that thioredoxin could suppress the 1-methyl-4-phenylpyridinium ion-induced ER stress by inhibiting the activation of IRE1α, TRAF2, JNK, caspase-12, and CHOP [30].

Therefore, we postulated that the inhibitory effect of losartan on the ER stress might be mediated through the induction of HO-1 and thioredoxin. We found that losartan increased the expression of HO-1 and thioredoxin, and also that the inhibitor of HO-1 and thioredoxin blocked the inhibitory effect of losartan on the tunicamycin-induced ER stress in tubular epithelial cells, thereby suggesting that the inhibitory effect of losartan on the ER stress was mediated through the induction of HO-1 and thioredoxin. In line with our results, it has been reported that the inhibition of 6-hydroxydopamine-mediated ER stress-induced apoptosis by insulin-like growth factor-1 was mediated through the regulation of HO-1 in PC12 cells [15]. Similarly, it has been suggested that the inhibition of tunicamycin- or thapsigargin-induced ER stress by AMP-activated protein kinase was mediated via the induction of HO-1 and thioredoxin [14].

It is well known that SIRT1 is able to induce the expression of HO-1 by the activation of Nrf2 as well as thioredoxin through FoxO3a and PGC-1α [31]. Therefore, we postulated that the inhibitory effect of losartan on ER stress might be mediated through the up-regulation of SIRT1, followed by the induction of HO-1 and thioredoxin. We found that treatment with SRT1720 (SIRT1 inducer) induced the expression of HO-1 and thioredoxin, which were prevented by sirtinol (SIRT1 inhibitor). Sirtinol also blocked the losartan-induced expression of HO-1 and thioredoxin, suggesting that the inhibitory effect of losartan on the ER stress was mediated through the SIRT1-HO-1/thioredoxin pathway.

Epithelial mesenchymal transition (EMT) is a process in which primary epithelial cells lead to morphological changes to fibroblastoid morphology, the up-regulation of mesenchymal markers including α-smooth muscle actin, vimentin, and fibroblast-specific protein-1 and the down-regulation of epithelial markers such as E-cadherin, ZO-1, and cytokeratin [32]. It has been suggested that renal tubular epithelial cells are able to become fibroblasts through EMT and participate in the pathogenesis of tubulointerstitial fibrosis [33].

ER stress is also a known cause of EMT and we previously reported that ER stress was able to induce EMT in renal tubular cells [34]. In this study, we found that losartan could suppress the tunicamycin-induced EMT as well. Consistent with our results, it has been reported that losartan suppresses the high-glucose-induced EMT in renal proximal tubular cells [35].

It is well proven that losartan ameliorates renal fibrosis in the animal model of 5/6 nephrectomy, unilateral ureteral obstruction, diabetic nephropathy, ischemia-reperfusion injury, and cyclosporine-induced fibrosis [36,37,38,39,40]. In this study, we found that sirtinol (SIRT1 inhibitor) could block the inhibitory effect of losartan on the UUO-induced renal fibrosis, suggesting that the anti-fibrotic function of losartan might be mediated at least in part through the inhibition of ER stress via the up-regulation of SIRT1.

ER stress is known to play important roles in renal pathology during nephrotoxicity, ischemia-reperfusion injury, viral infections, glomerulonephritis, podocytopathies, albuminuria, and kidney ageing [41]. Therefore, our data suggest that inducing SIRT1, HO-1, and thioredoxin may have clinical therapeutic potential in kidney diseases under excessive ER stress conditions.

## 4. Materials and Method

### 4.1. Reagent

Tunicamycin, sirtinol, PX12, and fatty acids-bearing albumin (A4919, low endotoxin <0.1 ng/mg) were obtained from Sigma Chemical Company (St. Louis, MO, USA). TGF-β, angiotensin II and glucose were obtained from R&D Systems (Minneapolis, MN, USA). Antibody to GRP78 was acquired from Santa Cruz Biotechnology (Santa Cruz, CA, USA). Antibodies to heme oxygenase-1, thioredoxin, total eIF2α (eukaryotic translation initiation factor-2α), phosphospecific-eIF2α (Ser^51^), α-smooth muscle actin, E-cadherin, and horseradish peroxidase-conjugated secondary antibody were purchased from Cell Signaling Technology (Beverly, MA, USA). Zinc protoporphyrin IX (Zn(II)PPIX) was obtained from Calbiochem (San Diego, CA, USA).

### 4.2. Cell Culture and Conditioning

All experiments were performed using HK-2 cells, a human proximal tubular cell line [42].HK-2 cells were obtained from the American Type Culture Collection. The media were changed every three days until confluent. Cells were growth-arrested in serum-free medium for 24 h before being used in experiments. To determine whether losartan suppressed the tunicamycin (TM)-induced ER stress, cells were incubated with TM (0.2 µM) with or without losartan (10 µM) for 24 h. To determine whether losartan suppressed the TGF-β-, angiotensin II-, high glucose-, and albumin-induced ER stress, cells were incubated with TGF-β (10 ng/mL), angiotensin II (1 µM), high glucose (30 mM), and albumin (5 mg/mL) for three days and then treated with losartan (10 µM) for two days. Concentrations of TM, TGF-β, angiotensin II, high glucose, and albumin used in our experiments were based on a previous study [14].

### 4.3. Western Blot Analysis

Proteins of whole-cell lysates were fractionated by 10% SDS polyacrylamide gels, and transferred onto a nylon membrane. The membranes were blocked with Tris-buffered saline containing 5% non-fat milk, probed with primary antibody for 2 h, followed by peroxidase-conjugated secondary antibody. The blots were developed by using enhanced chemiluminescence system (Amersham Pharmacia Biotech, Arlington, IL, USA). The band intensities were quantified by using a GS-710 densitometer and Quantity One software (version 4.2.1, Bio-Rad, Hercules, CA, USA). The results were normalized to β-actin as an internal control for standardization.

### 4.4. Experimental Mouse Model of Tunicamycin-Induced ER Stress

Male mice (C57BL/6), weighing about 20 g, were kept on a 12 h light/dark cycle in a temperature-controlled room, with free access to water and standard chow. A mouse model of tunicamycin-induced ER stress was induced by a single intraperitoneal injection of tunicamycin (TM, 2 mg/kg). In this model, it has been reported that male mice show high induction of ER stress markers such as GRP78 and CHOP with proximal tubular damages in the outer cortex [43]. Mice were randomly divided into four groups: control mice (*n* = 4), mice with TM injection (TM mouse, *n* = 4), TM mice with losartan (*n* = 4), and TM mice with losartan plus sirtinol (5 mg/kg i.p.) (*n* = 4). Losartan was administered by gavage at 10 mg/kg/day. Mice were euthanized after three days.

### 4.5. Experimental Mouse Model of Unilateral Ureteral Obstruction (UUO)-Induced Progressive Kidney Injury

UUO model is characterized by interstitial inflammatory cell infiltration, oxidative stress, apoptosis, and fibrosis. It has been known that angiotensin II and TGF-β contribute to UUO-induced renal fibrosis [44]. Furthermore, it has been reported that activation of ER stress is associated with UUO-induced renal apoptosis and fibrosis [18]. Therefore, we used a mouse model of UUO to test the hypothesis that the anti-fibrotic effect of losartan was mediated through the inhibition of ER stress via up-regulation of SIRT1. For induction of unilateral ureteral obstruction, male mice (C57BL/6) weighing about 20 g were anesthetized using evertin (125 mg/kg) and the left ureter was ligated through flank incision. Mice were randomly divided into four groups: control mice (*n* = 4), mice with UUO (*n* = 4), mice with UUO plus losartan (*n* = 4), and mice with UUO plus losartan and sirtinol (5 mg/kg i.p.) (*n* = 4). Losartan was administered by gavage at 10 mg/kg/day. On the 14th day after UUO surgery, the mice were sacrificed by using CO_2_. The kidney was fixed in 4% buffered formalin and embedded in paraffin for histological evaluation. Tubulointerstitial fibrosis was assessed by degree of interstitial collagen deposition using Masson trichrome stain [18]. All animal protocols were approved by Institutional Animal Care and Use Committee of Asan Institute for Life Sciences.

### 4.6. Immunohistochemical Staining

Paraffin embedded tissues were cut into 4 um sections and were deparaffinized in xylene and rehydrated in graded ethanol. To block endogenous peroxidase activity, sections were immersed in 0.3% hydrogen peroxide in PBS (phosphate-buffered saline) for 30 min. A microwave-based antigen retrieval method with 10 mmol/L citrate buffer (pH 6.0) for 10 min was done.

Nonspecific binding was blocked in 1% BSA (bovine serum albumin). Sections were then incubated with primary antibodies for GRP78, HO-1, and thioredoxin for 2 h and biotinylated secondary antibodies for 1 h and horseradish peroxidase-streptavidin conjugate for 30 min, followed by detection using DAB (3,3’-diaminobenzidine) stain (Dako, Glostrup, Denmark). The sections were counterstained with hematoxylin.

### 4.7. Statistical Analysis

Data were expressed as mean ± S.E. Statistical analysis was performed using Kruskall-Wallis, followed by a Mann-Whitney *U*-test using SPSS for Window 10.0 (SPSS Inc., Chicago, IL, USA). *p* < 0.05 value was considered statistically significant.

## 5. Conclusions

In conclusion, our study provides new information about the renoprotective effects of losartan, which may serve as the foundation for the targeted therapies designed to reduce ER stress.
